# Systematic Prediction of Pharmacodynamic Drug-Drug Interactions through Protein-Protein-Interaction Network

**DOI:** 10.1371/journal.pcbi.1002998

**Published:** 2013-03-21

**Authors:** Jialiang Huang, Chaoqun Niu, Christopher D. Green, Lun Yang, Hongkang Mei, Jing-Dong J. Han

**Affiliations:** 1 Key Laboratory of Computational Biology, Chinese Academy of Sciences-Max Planck Partner Institute for Computational Biology, Shanghai Institutes for Biological Sciences, Chinese Academy of Sciences, Shanghai, China; 2 Center for Molecular Systems Biology, Institute of Genetics and Developmental Biology, Chinese Academy of Sciences, Beijing, China; 3 Bioinformatics, Integrated Platform Science, GlaxoSmithKline Research and Development China, Shanghai, China; 4 Systematic Drug Repositioning, Computational Biology, GlaxoSmithKline, Philadelphia, Pennsylvania, United States of America; University of Heidelberg, Germany

## Abstract

Identifying drug-drug interactions (DDIs) is a major challenge in drug development. Previous attempts have established formal approaches for pharmacokinetic (PK) DDIs, but there is not a feasible solution for pharmacodynamic (PD) DDIs because the endpoint is often a serious adverse event rather than a measurable change in drug concentration. Here, we developed a metric “S-score” that measures the strength of network connection between drug targets to predict PD DDIs. Utilizing known PD DDIs as golden standard positives (GSPs), we observed a significant correlation between S-score and the likelihood a PD DDI occurs. Our prediction was robust and surpassed existing methods as validated by two independent GSPs. Analysis of clinical side effect data suggested that the drugs having predicted DDIs have similar side effects. We further incorporated this clinical side effects evidence with S-score to increase the prediction specificity and sensitivity through a Bayesian probabilistic model. We have predicted 9,626 potential PD DDIs at the accuracy of 82% and the recall of 62%. Importantly, our algorithm provided opportunities for better understanding the potential molecular mechanisms or physiological effects underlying DDIs, as illustrated by the case studies.

## Author Summary

Drug-drug interaction (DDI) is an important problem in clinical practice. In this study, we developed a novel algorithm for systematically predicting pharmacodynamic (PD) DDIs through protein-protein-interaction (PPI) networks. We calculated a score to predict potential PD DDIs by integrating the information from drug-target associations, PPI network topology and cross-tissue gene expression correlations. The scoring system was validated by known PD DDIs and agreed with similarities in drug clinical side effects, which we further integrated to increase the prediction performance. Our approach not only outperformed previously published methods in predicting DDIs, but also provided opportunities for better understanding the potential molecular mechanisms or physiological consequences underlying DDIs.

## Introduction

Drug-drug interaction (DDI) is a significant cause of adverse drug reactions (ADRs), especially in patient populations on multiple medications. A recent study indicated that medications were commonly used together in older adults, with nearly 1 in 25 individuals potentially at risk of a major DDI [1]. Approximately 70% of interactions are clinically relevant and contribute to the majority of ADRs [2]. DDIs occur when the pharmacologic effect of a given drug is altered by the action of another drug [3], leading to unpredictable clinical effects. DDIs can be categorized into three types: pharmaceutical, pharmacokinetic (PK), and pharmacodynamic (PD) [4], [5]. Pharmaceutical interactions occur because of a physical or chemical incompatibility. A PK interaction occurs when one medication alters the absorption, distribution, metabolism, or excretion of another, changing the drug concentrations arriving at the target sites. PD interactions occur if one drug has an antagonistic, additive, synergistic or indirect pharmacologic effect on another.

Current studies mainly focused on PK (especially Cytochrome P450 enzymes) DDIs and established experimental and simulation approaches to test for metabolic or transporter-based drug interactions [6]. However, a large number of DDIs cannot be explained at the PK or pharmaceutical levels and are supposed to be potential PD DDIs (Figure S1, Materials and Methods). Many of these interactions are not easily discernible because the endpoint is often a potentially serious adverse event rather than a measurable change in the concentration of the drug [5]. Typically, the potential PD DDIs were mainly based on sporadic cases reported during clinical trials. A number of severe PD DDIs are not identifiable in the early stage and result in great losses to human health. Thus far, the computational solutions to predict DDIs have used two distinct approaches. The first approach, termed similarity-based, predicted DDIs by measuring the similarity of drug information. As an example, Gottlieb *et al* [7] utilizes multiple drug-drug similarity measures to predict DDI. In this respect, many previous methods which were originally designed for inferring novel potential targets of drugs based on various types of data, such as structures [8], targets [9], indications [10], side-effects [11] and gene expression profiles [12], can also be used to infer drug interactions. The second approach is the knowledge-based approach that predicts DDI from scientific literature [13], an electronic medical record database [14] and the FDA Adverse Event Reporting System [15]. However, both approaches suffer from several limitations, such as the necessity to distinguish drug classes and the inability to handle novel drugs for which limited reports exist [7]. More importantly, they seldom consider drug actions and their clinical effects in the context of complex biological networks.

To ameliorate this situation, we adopted a network pharmacology strategy [16], which considers drug actions and their clinical effects in the context of molecular network systems, and proposed an algorithm to systematically predict PD DDIs. Using known PD DDIs as golden standard positives (GSPs), we demonstrated the superiority of our algorithm over previously published methods. The predictions also agreed with similar clinical side effects between the drugs, which was further incorporated with S-score to increase the prediction performance through a Bayesian probabilistic model. Importantly, our methods provided not only a comprehensive list of potential PD DDIs, but it also opportunities for further understanding of the molecular mechanism and physiological effect underlying DDIs.

## Results

### PD DDIs are reflected at the molecular network level

**Figure 1:**
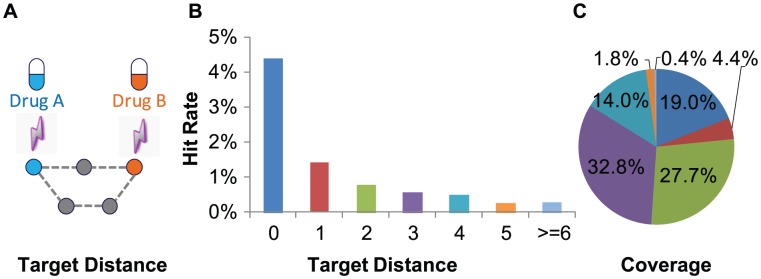
PD DDIs reflected at the molecular network level. (**A**) A representative example of drug target distance: the minimum distance of the shortest path between two drugs' targets on PPI network. (**B–C**) Hit rate (**B**) and coverage(**C**) of known PD DDIs among the drug pairs grouped by their target distances on the PPI network.

To determine whether the network pharmacology strategy can be used to understand DDIs, specifically PD DDIs, we first investigated whether PD DDIs are reflected at the network level. We examined the distribution of the targets of drug pairs with known PD DDIs among the 1,249 FDA-approved drugs collected in DrugBank [17] in a protein-protein interaction (PPI) network from HPRD [18]. The connection for any possible drug pairs was measured by the minimum shortest path between their targets in the PPI network (Figure 1A, Materials and Methods). Out of the 21,049 drug pairs that have minimum target distances of zero, 924 (4.4%) were known PD DDIs (Figure 1B), which represents a ∼6-fold enrichment compared with all possible drug pairs. This is expected because drug pairs with minimum distances of zero are those sharing at least one overlapping target, and these have been reported to have a high probability to form DDIs [9]. More importantly, we found that the smaller the minimum distance between two drugs' targets the more likely a PD DDI occurs (Figure 1B), suggesting PD DDIs can be discerned at the PPI network level. In fact, the drug pairs with the minimum distance ≤3 already cover the majority (>80%) of the known PD DDIs (Figure 1C). Overall, the average distance of known PD DDI targets is significantly shorter than the global average of possible drug pairs in the network (*P-value<2.2E-16*, Wilcoxon rank sum test).

### An algorithm for predicting PD DDIs through PPI network

**Figure 2:**
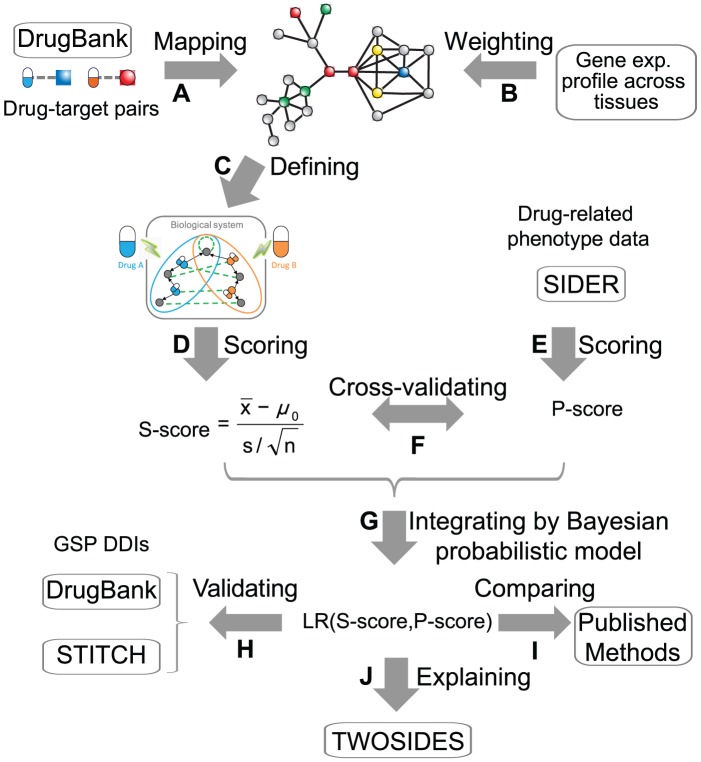
Algorithmic pipeline. (**A**) Mapping drugs onto PPI network through drug-target associations. (**B**) Weighting PPI network by gene expression profile across human tissues. (**C**) Defining target-centered system, including known drug-targets and their first-step neighbor protein on PPI network. (**D**) Scoring systematic connectivity based on drugs' target-centered systems (S-score). (**E**) Scoring phenotypic similarity based on drug clinical side effects (P-score). (**F**) Cross-validating between S-score and P-score. (**G**) Integrating by a Bayesian probabilistic model. (**H**) Validating the prediction performance using GSP DDIs from two independent databases, DrugBank and STITCH. (**I**) Comparing with other methods. (**J**) Explaining the molecular mechanisms of the polypharmacy side effects recorded in TWOSIDES.

Based on the above observation, we designed a metric for systematically predicting PD DDIs by considering drug actions in the context of the PPI networks. First, drugs were mapped onto a PPI network based on their drug-target associations (Figure 2A). Second, many drugs exert their therapeutic or adverse effects by interfering with tissue-specific molecular targets that are usually located in the same tissue where a pathological process occurs [19]. Therefore, we weighed the PPI in the network by Pearson's correlation coefficient (PCC) of their encoding genes' expression profile across 79 human tissues [20] (Figure 2B). Then we defined a target-centered system for each drug, which includes drug targets and their first-step neighboring proteins in the PPI network (Figure 2C). Finally, we defined a system connection score (S-score) to describe the connection between two target-centered systems in the PPI network as the following (Figure 2D):
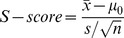
where 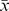,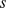 and 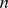 represent the mean, standard deviation and number of the cross-tissue expression PCC of edges connecting two drug-centered systems, respectively; 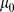 represents the average PCC of all edges in the network as background. In addition, if two target-centered systems share a gene, an artificial edge with PCC of 1 is added between the two systems. Thus, S-score reflects the tightness of connection between two target-centered systems in the network, which not only depends on the number of edges connecting the genes in these two target-centered systems but also on the similarity in expression patterns across tissues.

### Performance evaluation and comparison with other methods

**Figure 3:**
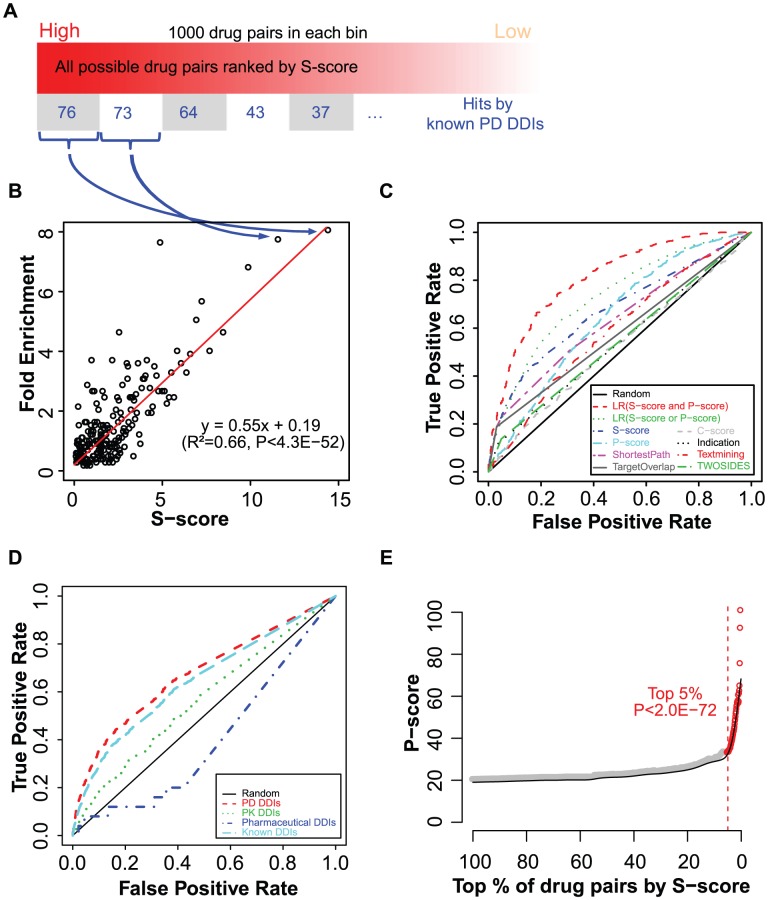
Performance evaluations. (**A**) All possible drug pairs ranked and binned into groups by their S-score. (**B**) Fold-enrichment of hits of known PD-like DDIs in each bin (*y*-axis) plotted against average S-score (*x*-axis). (**C**) ROC curves to evaluate the performance of various scoring methods using DrugBank PD-like DDIs as GSPs. (**D**) ROC curves to evaluate the prediction performance of S-score using three types (PD, PK, pharmaceutical) or known DDIs in DrugBank as GSPs. (**E**) Associations of S-score with drug clinical side effect similarity (P-score). Average of P-score (y-axis) is plotted for top N% of drug pairs ranked by S-score (x-axis). *P-value* for top 5% drug pairs against all possible drug pairs was calculated from Wilcoxon rank sum test.

To evaluate our scoring scheme, we calculated S-scores for all possible drug pairs among the list of FDA-approved drugs. Using known PD DDIs collected in DrugBank as GSPs, we first evaluated the correlation between S-score and the likelihood that a PD DDI occurs. Indeed, the occurrence of PD DDIs decreased with decreasing S-scores among all possible drug pairs (Figure 3A). Additionally, there was a highly significant correlation between S-score and the hits enrichment of GSPs (*R^2^ = 0.66, P-value = 4.3E-52*) (Figure 3B). It indicated that the likelihood of a PD DDI to occur is high if the two drugs' targets are highly connected in PPI network and co-expressed in the same tissues. Next, we used receiver operating characteristic (ROC) curves to examine the performance of our algorithm. We compared our prediction with previously published methods (Materials and Methods, Text S1): (1) target overlap, connecting two drugs if share at least one target [9]; (2) target distance, connecting two drugs by their minimum distance of shortest path between targets on PPI network (Materials and Methods); (3) P-score, connecting two drugs by their side-effect similarities [11]; (4) C-score, connecting two drugs by their gene-expression signatures connectivity [12]; (5) indication overlap, connecting two drugs if they share a similar indication [10]; (6) text mining, connecting two drugs based on a co-occurrence scheme [13]; (7) TWOSIDES, a database of polypharmacy side effects for pairs of drugs mined from FDA Adverse Event Reporting System [15]; (8) INDI, a method predicted DDIs utilizing multiple drug-drug similarity measures [7]. Targets-based methods (target overlap, target distance and S-score) are better than those using indication, gene-expression signatures or side-effect similarities to connect drugs (Figure 3C). Importantly, S-score, by integrating the information from drug-target associations, PPI network topology and cross-tissue gene co-expression, has the best performance (Figure 3C). Interestingly, using different types of known DDIs as GSPs (Materials and Methods), we found that S-score mainly predicted PD, but not PK or pharmaceutical DDIs (Figure 3D). Using the DDIs recorded in DrugBank as GSPs, we also observed that our method outperformed previous methods (Figure S2A). By using the drug-drug associations with medium text mining confidence score from the STITCH database [13] as another evaluation criterion, we also confirmed the robustness of S-score in predicting potential DDIs (Figure S2B), even for our novel predictions which excluded the known DDIs in DrugBank (Figure S2C). These results excluded the possibility that the performance of S-score was associated with biases of our semi-automatic text-mining method of classifying known DDIs into three types, and demonstrated the good performance of S-score is independent of the GSPs used. Expectedly, taking a negative set with a different size had a negligible effect on the result (Figure S2D).

### Drugs having predicted DDIs produce similar side effects

To further validate our predictions, we examined the phenotypic effects of our predictions using published drug clinical side effect data [21]. Based on the observation that similar disorder phenotypes indicate overlapping molecular mechanisms [22], we asked whether two drugs have similar clinical outcomes if they are highly connected in their target-centered systems (Figure 2E and 2F). We measured the phenotypic connections between two drugs by their side-effect similarities (P-score) following a published algorithm, which was originally designed to infer novel potential targets of marketed drugs [11]. The drug pairs with high S-scores indeed had more similar phenotypes (Figure 3E, *P* *-value = 2.0E-72*, Wilcoxon rank sum test). Thus, S-score calculated using PPI network might partially explain the drug phenotypic overlap.

### Integration by a Bayesian probabilistic model

To further increase the prediction performance, we integrated the evidences from S-score and P-score as a likelihood ratio (LR) using a Bayesian probabilistic model (Figure 2G, Materials and Methods). As a result, we observed a clear improvement of prediction specificity and sensitivity (Figure 3C). The area under the ROC curve (AUC) increased from 0.674 to 0.731. In particular, for drug pairs with both evidences, the AUC of LR (defined as LR(S-score and P-score)), approached 0.812 (Figure 3C). We applied the algorithm to the FDA-approved drugs and generated a list of prioritized drug pairs where PD DDIs might likely occur. Overall, the list of 9,626 drug pairs with LR(S-score and P-score) >2 were 7.5-fold enriched for known PD DDIs against all possible drug pairs (Table S1), which represents an accuracy of 82% and a recall of 62% (Materials and Methods). To further assess our novel predictions, we evaluated the potential side effects of our novel predictions against the TWOSIDES database [15], which collected polypharmacy side effects for pairs of drugs from the FDA Adverse Event Reporting System (Figure 2J). We observed a significant overlap between our novel predictions and TWOSIDES (*P-value*<2.2E-16, Fisher's exact test), where 27% of the novel predictions overlapped the list of TWOSIDES. The percentage approached 60% for our top 100 novel predicted drug pairs (Table S1). The prioritized list together with the available drug indication information, such as whether two drugs were likely co-used, can provide the rationale for which PD DDIs we should be mindful of during clinical trials or treatment.

### Case studies

**Figure 4:**
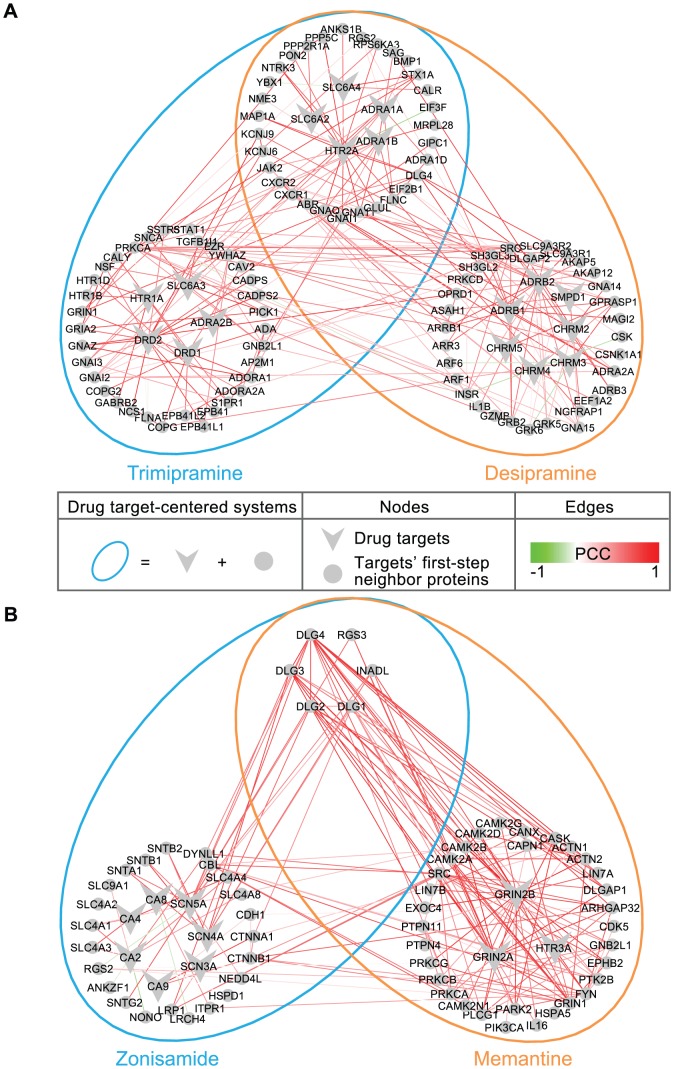
Case studies. (**A**) Desipramine and trimipramine. (**B**) Zonisamide and memantine. The drug target-centered systems (ellipse) colored for different drugs, includes drug targets (vee) and their first-step neighboring proteins (circle) in the PPI network. The edges in the network were weighted by the PCC based on cross-tissue expressions.

The most common drugs at the top of the prioritized list of potential PD DDIs were associated with tricyclic antidepressants (TCA) (Table S1), which are primarily used in the clinical treatment of mood disorders such as major depressive disorder (MDD) and dysthymia. It has been reported that patients taking antidepressants have more opportunities to experience DDIs, because antidepressants are often prescribed for months or years. In addition, patients with depressive disorders typically have comorbid symptoms that require administration of concomitant medications [23]. Although many of these drug interaction mechanisms remain unclear, it is recommended that concomitant therapy of TCAs should be used with caution considering the major clinical significance [24], [25]. As an example, within the top 10 predicted DDIs, a potential interaction was predicted between two TCAs (desipramine and trimipramine) (Table S1). Such an interaction has been reported to increase the risk of additive QTc-prolongation and serious ventricular arrhythmias in DrugBank [17]. In our network model, the target-centered systems of these two drugs highly overlapped and connected with correlated cross-tissue gene expression (Figure 4A), which is indicated by an S-score of 9.6 (Student's *t*-test *P-value<2.2E-16*, compared to all possible drug pairs). Interestingly, both of two drugs' target-centered systems are enriched in genes associated with the Gene Ontology “regulation of heart contraction” (GO:0008016) (*P-value* = 6.9E-5 and *P-value* = 1.6E-3, respectively), which might help in explaining the molecular basis of the potential outcome of the concomitant administration of the two drugs.

Our novel predictions together with the information from TWOSIDES provided opportunities for better understanding the potential molecular mechanisms or physiological effects underlying DDIs (Figure 4B, Figure S3 and Text S1). As an example, an interaction was predicted to exist between zonisamide and memantine (Figure 4B). Zonisamide is a sulfonamide anticonvulsant approved for using as an adjunctive treatment of partial seizures in adults with epilepsy by blocking sodium and calcium channels, which leads to the suppression of neuronal hypersynchronization (i.e. convulsions) [17]. Memantine, an amantadine derivative used in the treatment of Alzheimer's disease, exerts its action through uncompetitive NMDA receptor antagonism, which protects against elevated concentrations of synaptically released glutamate in the brain of demented patients [17]. The two drugs do not have common targets, but do have similar cross-tissue expressions between their drug-centered systems (S-score = 6.5, *P-value*<2.2E-16) and similar side effects (P-score = 73.5, *P-value*<2.2E-16). Although it has not been reported in DrugBank [17], TWOSIDES recently reported that this drug pair has an significant association with the adverse event thrombocytopenia (*P-value* = 1.36E-177) in the FDA Adverse Event Reporting System, which cannot be clearly attributed to the individual drugs alone [15]. Our analysis reveals that the genes in two drug target-centered systems are highly enriched in genes significantly highly expressed in the “Platelet” (UP_TISSUE) (*P-value* = 8.8E-3). Interestingly, such an interaction cannot be predicted based only on the knowledge of their drug targets as neither of the individual drug's target gene set is related to the thrombocytopenia symptom. Yet, consistent with their intended effects, emantine's targets are enriched for “N-methyl-D-aspartate selective glutamate receptor complex” (GO:0017146) (*P-value* = 1.4E-2), which is involved in Alzheimer's disease [26], while zonisamide's targets are enriched for “voltage-gated sodium channel complex” (GO:0001518) (*P-value* = 3.1E-4), which is involved in pathological alterations in epilepsy [27].

Additional examples of novel predictions of the PD DDIs can be found in Figure S3 and Text S1.

## Discussion

Despite the many methods previously applied to identify potential drug interactions from different aspects, these approaches have various limitations. To our knowledge, we for the first time, present an algorithm for systematically predicting PD DDIs by considering drug actions and their clinical effects in the context of complex PPI networks. The integration of various sources of information such as drug targets, network topology, cross-tissue gene expression correlations and side effect similarity indeed give rise to a better performance in predicting DDIs than those obtained with individual data sources. Finally, our network model provides opportunities for better understanding the potential molecular mechanisms or physiological effects underlying DDIs.

However, like other computational-based techniques in this field, there still exists a gap between our scientific predictions in theory and clinical application. First, limited by the current knowledge of the molecular network as well as the robustness of the biological system itself, our prediction only provides the relative likelihood of the occurrence of a PD DDI. Second, as currently only a few types of data were used for prediction, the prediction power is bound to improve when integrated with more clinical data, if available, and complemented with recently published DDI prediction methods from different aspects. Last, the predicted potential PD DDIs are not necessarily always harmful but sometimes can also be beneficial [28]. Even though the current GSPs include only a small number of beneficial interactions, such interactions may occur through the same mechanism - overlapping network, in which case can be predicted by our method. With these further improvements, our method can be potentially applied in drug discovery and development, serving as an *in silico* systematic screen to provide a list of prioritized potential PD DDIs in a cost-effective manner or be applied to relabeling drug interaction warnings for marketed drugs. Our method can also reveal potential mechanisms or effects underlying DDIs and provide the necessary scientific evidence for further investigation of the drugs during clinical trials. These mechanisms could be valuable for rational poly-medication among existing drugs for new purposes to enhance beneficial drug combinations while avoiding harmful DDIs.

## Materials and Methods

### Data sets

Drug information was downloaded from DrugBank database (http://www.drugbank.ca/) on May 9, 2011. In DrugBank, a drug target is defined as “a protein, to which a given drug binds, resulting in an alteration of the normal function of the bound molecule and a desirable therapeutic effect”. In our further analysis, we mainly focused on the list of 1,249 FDA-approved drugs which include 4,776 associations with 1,289 targets. A PPI network, including 34,998 edges, was taken from Human Protein Reference Database (HPRD; http://www.hprd.org/) [18] on Dec 7, 2010. To weight the edges in the network, we used PCC based on the pair-wise gene expression profiles in 79 human tissues [20].

### Prediction assessment

#### Golden Standard Positives (GSPs)/Negatives (GSNs)

To measure the performance of predicted drug interactions, 14,746 known DDIs from DrugBank which are defined as “interact, interfere or cause adverse reactions when taken with two drugs”, were used as GSPs. We separated these DDIs into three classes based on their clinical label descriptions using a semi-automatic text mining method: (1) pharmaceutical interactions, where the description label contains any of the keywords- “physicochemical”, “non-absorbable”, “solution”; (2) PK interactions, where the label contains any of the keywords- “CYP”, “absorption”, “distribution”, “metabolism”, “excretion”, “concentration”, “level”, “metabolite”, “metabolized”, “enzyme inducer”, “clearance”; (3) PD-like interactions - the rest. The classification has also been used in a paper recently published [7]. In total, it resulted in 82 pharmaceutical DDIs, 3,259 PK DDIs and 11,405 PD-like DDIs (Figure S1). In our analysis, we used the PD-like DDIs as GSPs if not stated otherwise. Since the true negative set of DDIs is not available, similar to a recent study [7], we randomly generated a set of drug pairs (not part of the GSPs) with five-times the size or an equal size as the GSPs, as the GSNs. The difference in the size of the GSNs has negligible effect on the result (Figure 3C and S2D). Therefore, we used GSNs with an equal size as the GSPs if not stated otherwise.

To demonstrate the robustness of our method in predicting potential DDIs, 32,403 drug-drug associations (among the drugs listed in DrugBank) with medium text mining confidence score (>400) from STITCH database (http://stitch.embl.de/) were used as another evaluation criterion.

#### Fold enrichment

To evaluate whether the drug pairs identified by high S-score are more likely to occur GSP DDIs, all possible drug pairs were ranked by the S-score and binned into groups of 1,000 drug pairs. Fold enrichment over background is defined as (*m/n)/(M/N*), where *m* is the number of GSPs among all possible *M* GSPs within each bin of *n* drug pairs among the total of *N* all possible drug pairs.

#### ROC curve

We used ROC curves to evaluate the performance of our prediction. True positive rate (TPR) and false positive rate (FPR) are defined as:
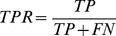


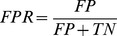
where *TP* represents true positive, *TN* represents true negative, *FP* represents false positive, *FN* represents false negative.

#### Accuracy and recall

For a drug pair with both evidences from S-score and P-score, we used LR(S-score and P-score) >2 as the threshold to determine whether a PD DDI might actually occur between the two drugs. Accuracy and recall are defined as:
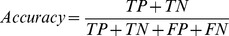


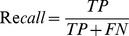



### Comparison with other methods

To compare the prediction performance of our algorithm with previously published methods, we selected several representative methods in the field of DDI prediction [7], [13], [15] and also covered some approaches that were originally designed for inferring novel potential targets of drugs but can also be used to infer drug interactions [8], [9], [10], [11].

#### Target overlap

Yildirim *et al* constructed a ‘drug network’, with nodes representing drugs, and two drugs connected to each other if they share at least one target protein [9].

#### Target distance

Instead of simply calculating the overlap between two drugs' targets, we measured the connection between drug targets by their minimum shortest distances in PPI network. Shortest path between any two targets was first searched using Dijkstra's algorithm. Then, we selected the minimum distance of the shortest path between their targets to describe the distance of two drugs on PPI network (Figure 1A).

#### C-score

The expression connectivity score (C-score) of all possible drug pairs are locally calculated following the algorithms of Connectivity Map [12], which uses gene-expression signatures to connect small molecules based on the assumption that cells treated by drugs with similar mechanisms of action generally exhibit correlated gene expression profile. The drug, batch and gene expression information of all ∼6,000 drug treatments were downloaded from cMAP website (http://www.broadinstitute.org/cmap/) on May 27, 2011.

#### P-score

Campillos *et al* used side-effect similarities (P-score) to measure the connection between two drugs to infer novel molecular interactions between drugs and potential targets [11]. We calculated the phenotypic similarity following their algorithm by using a more comprehensive side effects resource of ∼800 drugs downloaded from a public side effect resource (http://sideeffects.embl.de/) that connects 888 marketed drugs to 1,450 side effect terms [21].

#### Indication overlap

In Berger and Iyengar's work, drugs were connected if sharing a common therapeutic indication described by the Anatomical Therapeutic Chemical Classification System (ATC) third level codes [10]. It is based on the notion that co-administration of drugs for a common indication may enhance clinical overlapping outcome, which is moderate when given alone. The drug and ATC-code mapping was downloaded from DrugBank on May 9, 2011.

#### Text mining

The text mining score between two drugs indicating their association based on a co-occurrence scheme were downloaded from the STITCH database [13] (http://stitch.embl.de/) on June 11^th^, 2012.

#### TWOSIDES

TWOSIDES is a database of polypharmacy side effects for pairs of drugs mined from FDA Adverse Event Reporting System [15]. It contains 868,221 significant associations between 59,220 pairs of drugs and 1,301 adverse events. Drugs were connected if they are associated with at least one adverse event that cannot be clearly attributed to the individual drugs alone.

#### INDI

Gottlieb *et al* [7] predicted 46,709 non- cytochrome P450 (CYP)–related DDIs utilizing multiple drug-drug similarity measures, named INferring Drug Interactions (INDI).

### Bayesian probabilistic model

To integrate the evidences from network system connectivity score (S-score) and drug phenotypic similarity score (P-score), we used a Bayesian probabilistic model described in Xia *et al* [29], where the Bayesian model has been proven to be particularly competent in predicting PPIs by integrating various evidences. The method has also been used to combine the different types of clues for predicting PPIs in a paper recently published [30]. Briefly, in the Bayesian probabilistic model, each score is automatically weighted according to its confidence level. The scoring schemes (*S-score, P-score*) were integrated as a likelihood ratio (LR) for drug pairs to be true positive DDIs versus true negative DDIs by multiplying from all the independent evidences as following:
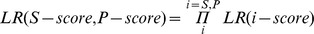


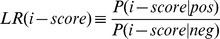
where *LR(i-score)* represents the likelihood ratio of evidence *i-score*. It relates prior and posterior odds according to the Bayes rule:
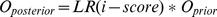


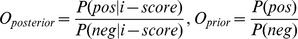
where the terms *‘posterior’* and *‘prior’* refer to the condition before and after considering the evidence information *i-score*; the *prior odds (O_prior_)* of finding the positive and negative hits can be can be calculated by considering the total number of GSP/GSN DDIs within all the possible drug pairs; the *posterior* odds (*O_posterior_*) can be calculated by binning all possible drug pairs into discrete intervals according to the evidence *i-score*. We defined LR (S-score and P-score) for drug pairs with both evidences, while LR (S-score or P-score) for those with at least one evidence, respectively.

### Functional annotation analysis

Functional annotation analysis was performed using the DAVID web-server [31].

### Availability

The datasets used in this paper and the core code in calculating the S-score were packaged and provided on our website http://www.picb.ac.cn/hanlab/DDI.

## Supporting Information

Figure S1 Three types of known DDIs curated in DrugBank. (**A**) Classification of DDIs in DrugBank using a semi-automatic text mining method. (**B**) Percentage of three types of known DDIs curated in DrugBank.
(EPS)

Figure S2 ROC curves to compare the prediction performance of various scoring methods. (**A**) GSP DDIs from DrugBank known DDIs. (**B**) GSP DDIs from STITCH DDIs with medium text mining confidence score. (**C**) GSP DDIs from STITCH DDIs with medium text mining confidence score to evaluate novel predictions by each scoring method, which excluded the known DDIs in DrugBank. (**D**) GSP DDIs from DrugBank PD DDIs and GSN DDIs randomly selected 5-times as large as the positive set (Materials and Methods).
(EPS)

Figure S3 Additional case studies. (**A**) Atenolol and Meperidine and (**B**) Mirtazapine and Propranolol. The figure legend is the same as those in Figure 4.
(EPS)

Table S1 List of drug pairs with LR (S-score and P-score) >2.
(XLSX)

Text S1 Appendix with detailed comparison with the recently published methods and discussion on additional case studies of novel PD DDIs predicted.
(DOCX)
