# Supplementary material for: Systematic Prediction of Pharmacodynamic Drug-Drug Interactions through Protein-Protein-Interaction Network
Source: PLoS Comput Biol. 2013 Mar 21;9(3):e1002998. doi: 10.1371/journal.pcbi.1002998 (PMC3605053; doi:10.1371/journal.pcbi.1002998)
Supplement: Text S1 — Appendix with detailed comparison with the recently published methods and discussion on additional case studies of novel PD DDIs predicted. (DOCX) [file pcbi.1002998.s005.docx]

## Systematic Prediction of Pharmacodynamic Drug-Drug Interactions through Protein-Protein-Interaction Network

Jialiang Huang^1,2,3^, Chaoqun Niu^1,2^, Christopher D. Green^1^, Lun Yang^4^, Hongkang Mei^3,*^ and Jing-Dong J. Han^1,*^

**Comparison with the recently published methods**

We compared our methods with the methods that have been recently published to predict DDIs from different aspects. Gottlieb *et al* [1] predicted 46,709 non-CYP-related DDIs utilizing multiple drug-drug similarity measures (named INDI). Unfortunately, due to the intellectual property issue, we can only access their 18,601 novel predictions, which exclude the gold standard DDIs. For a fair comparison, we also excluded the known DDIs in DrugBank from our predictions and found our predictions have better performance when using drug-drug associations with medium text mining confidence score (>400) from the STITCH database as GSPs (Figure S2C). Tatonetti *et al* [2] provided a database, TWOSIDES, including 868,221 significant associations between 59,220 pairs of drugs and 1,301 adverse events mined from the FDA Adverse Event Reporting System. Drugs were connected if they are associated with at least one adverse event that cannot be clearly attributed to the individual drugs alone. Using their method on our data set, we found that our method is better than TWOSIDES in prediction performance (Figure 3C and S2).

**Discussion on additional case studies of novel PD DDIs predicted.**

Figure S3A showed an interaction we predicted to exist between atenolol and meperidine. Atenolol, a competitive beta(1)-selective adrenergic antagonist, is approved for the management of hypertention and long-term management of patients with angina pectoris [3]. Meperidine, used to control moderate to severe pain, is primarily a kappa-opiate receptor agonist, and also a glutamate receptors agonist [3]. The two drugs do not have common targets or similar side effects (P-score=12.6, *P-value=1*), but do have similar cross-tissue expression between their drug-centered systems (S-score=10.7, *P-value*<2.2E-16). Genes in either of two drug target-centered systems are over-represented in Gene Ontology “[synaptic transmission](http://www.ebi.ac.uk/QuickGO/GTerm?id=GO:0007268)” (GO:0007268) (*P-value*=6.7E-3 and *P-value*=3.5E-3, respectively). Genes in these two drug target-centered systems are highly enriched for genes significantly highly expressed in the “Brain” (UP_TISSUE) (*P-value*=1.2E-4). This is consistent with the associated side effect neuropathy reported with this drug pair in TWOSIDES (*P-value*=6.36E-278) [2].

Figure S3B showed an interaction we predicted to exist between mirtazapine and propranolol. Mirtazapine, approved for the treatment of major depressive disorders, acts as an antagonist at the central pre-synaptic alpha(2)-receptors, inhibiting negative feedback to the presynaptic nerves and causing an increase in norepinephrine release [3]. Propranolol, used for the prophylaxis of migraine, competes with sympathomimetic neurotransmitters such as catecholamines for binding at beta(1)-adrenergic receptors in the heart, inhibiting sympathetic stimulation [3]. Although these two drugs do not share common targets, they both affect the G-protein coupled receptor signalling pathway (*P-value*=2.0E-4 and *P-value=*1.1E-8, respectively), which is involved in a wide variety of physiological processes. Co-usage of these two drugs have been reported to be associated with 202 adverse side effects in TWOSIDES [2].

1. Gottlieb A, Stein GY, Oron Y, Ruppin E, Sharan R (2012) INDI: a computational framework for inferring drug interactions and their associated recommendations. Mol Syst Biol 8: 592.

2. Tatonetti NP, Ye PP, Daneshjou R, Altman RB (2012) Data-driven prediction of drug effects and interactions. Sci Transl Med 4: 125ra131.

3. Knox C, Law V, Jewison T, Liu P, Ly S, et al. (2011) DrugBank 3.0: a comprehensive resource for 'omics' research on drugs. Nucleic Acids Res 39: D1035-1041.
